# Implementation research for promoting access and rational use of antibiotics for children: lessons learnt from Tanzania

**DOI:** 10.1093/jacamr/dlad045

**Published:** 2023-04-19

**Authors:** George M Bwire, Upendo O Kibwana, Lilian Nkinda, Betty A Maganda, Mathew Mganga, Arapha Bashir Nshau, Oliver Williams, Janet Midega, Elevanie Nyankesha, Robert W Scherpbier

**Affiliations:** Muhimbili University of Health and Allied Sciences, P.O. Box 65001, Dar es Salaam, Tanzania; Muhimbili University of Health and Allied Sciences, P.O. Box 65001, Dar es Salaam, Tanzania; Muhimbili University of Health and Allied Sciences, P.O. Box 65001, Dar es Salaam, Tanzania; Muhimbili University of Health and Allied Sciences, P.O. Box 65001, Dar es Salaam, Tanzania; President’s Office-Regional Administration and Local Government, P.O. Box 1923, Dodoma, Tanzania; Pharmacy Council, NHIF Building, 1st Floor, UDOM Road, P.O. Box 1277, Dodoma, Tanzania; Wellcome Trust, 215 Euston Road, London NW1 2BE, UK; Wellcome Trust, 215 Euston Road, London NW1 2BE, UK; United Nations Children’s Fund, New York Headquarter office, 3 United Nations Plaza, New York, NY 10017, USA; United Nations Children’s Fund, Bâtiment BIT, Route des Morillons 4, CH-1211, Geneva 22, Switzerland

## Abstract

Implementation research (IR) has proved to be a potential catalyst in facilitating the uptake of evidence-based innovations into routine practices and thereby maximizing public health outcomes. IR not only focuses on the effectiveness of the innovations but also identifies and addresses the barriers and facilitators to maximize their uptake into routine practices. This article describes the processes undertaken to implement a research project aimed at promoting access and rational use of antibiotics for children (PARAC). It also provides an overview of the lessons learnt during its implementation in Tanzanian hospital and community settings.

## Antimicrobial resistance (AMR)

AMR, especially resistant bacteria, is a public health threat that requires serious action to prevent the predicted post-antibiotic era.^1^ Globally, MDR bacteria cause 700 000 deaths across all ages, of which approximately 200 000 are children, especially neonates.^2^ Tanzanian patients with neonatal sepsis had around a 14% higher chance of dying with sepsis compared with those without sepsis. Drug-resistant bacteria have greatly contributed to this.^3^ A considerable number of evidence-based measures have been documented to curb the problem of AMR, but their successful implementation still remains a challenge.^4^ To address this, Tanzanian AMR stakeholders designed implementation research (IR),^5^ firstly to assess the implementation of stewardship programmes in 14 tertiary hospitals, and secondly to assess the contributions of accredited drug-dispensing outlets (ADDOs) to the access and rational use of medicines, particularly antibiotics.

## Steps for implementing the ‘promoting access and rational use of antibiotics for children’ (PARAC) project in Tanzania

**Figure 1.**
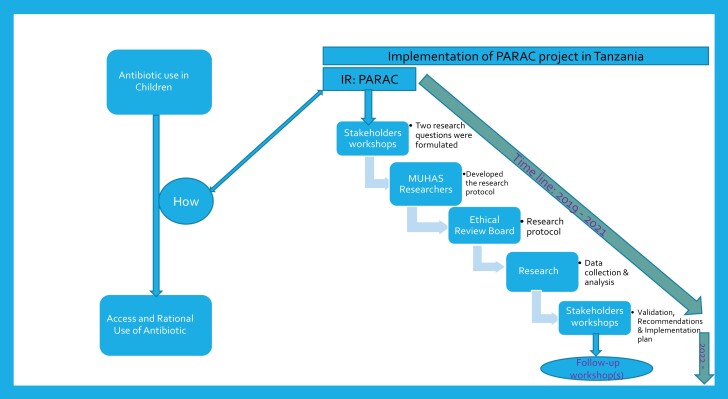
Timeline for implementation of the PARAC project in Tanzania.

This was a stakeholder-driven project, kicked off by UNICEF and funded by Wellcome where stakeholders were invited to discuss and formulate the research questions. Various stakeholders were engaged, from the government, the private sector, associations, non-government organizations (NGOs), multilateral agencies, academic agencies and beneficiaries.^6^ The implementation of the project for PARAC followed the steps as presented in Figure 1.

### Understanding the research gaps and formulation of the research questions

Stakeholders designed the research questions based on the implementation challenges and research gaps that were identified by the same stakeholders and evidence from literature and implementation experience. The formulation of the research questions was guided by the stakeholders with experience in research (e.g. research and academic institutions). Two research questions were formulated to target hospitals and community settings.

### Development of the study protocols

After having two approved research questions by stakeholders, the team of researchers from Muhimbili University of Health Sciences (MUHAS) had the role of developing the study protocols. From the two formulated questions, one protocol targeted the hospital settings (tertiary hospitals) and the other targeted the community settings, the so-called ADDOs. The two protocols were submitted to the MUHAS Research and Ethics Committee for ethical approval.

### Data collection and analysis

Data were collected from the 14 administrative regions (almost half of all regions) of Mainland Tanzania. A mixed-methods approach was used to study the questions designed by stakeholders. Before starting data collection, the approved protocols were submitted to the appropriate national and local authorities for granting of permissions. According to country regulations, ADDOs are regulated by the President’s Office—Regional Administration and Local Government. For the hospital-based study, permissions were requested from the medical officers in charge. The collected data were submitted to the experienced biostatisticians for quantitative analysis while for the interviews the qualitative research experts analysed the data. Two reports were prepared for dissemination to the funder and the stakeholders.

### Dissemination of the research findings

MUHAS researchers in collaboration with the Pharmacy Council (PC), prepared two research project reports. The reports were communicated and presented to the funder through the UNICEF country office for reviews and suggestions. The MUHAS team of researchers responded to the comments and suggestions. Following reviews from the funder reports, MUHAS and PC prepared workshops for stakeholders to validate the findings, formulate and prioritize actionable recommendations and to develop an action plan to include the recommendations into longer-term implementation plans. After the workshops, the research findings, actionable recommendations and their action plans were communicated to the Permanent Secretaries Ministry of Health and President’s Office—Regional Administration and Local Government.

### Formulation of actionable recommendations and their action plans

During the first workshop conducted in Dar es Salaam city, stakeholders spent 2 days validating the research findings and formulating actionable recommendations and the action plan. The participatory approach was used to formulate the actionable recommendations and the action plan from the validated findings. In this workshop, stakeholders reviewed the findings and developed actionable recommendations to address the gaps that were reported from the research findings. In the end, a plenary session was held where each of the actionable recommendations formulated by the groups were discussed. For both studies, the plenary session came up with 12 actionable recommendations plus their action plans.

### Prioritizing the actionable recommendations

About a month after the first workshop, stakeholders were invited to attend the second workshop in Dodoma city (Government city). The first workshop was researcher focused, and the second one was more policymaker focused on how these findings would translate into practice, and which findings had a greater likelihood of actually changing practice. The second workshop was organized after the team had time to share and discuss the recommendations from the first workshop with their representing organizations/institutions. During the second workshop, the actionable recommendations were prioritized and only six actionable recommendations were retained. For further actions, the six actionable recommendations were communicated to the Government Chief Medical Officer from the Ministry of Health and the Director of Health, Social Welfare and Nutrition Service—President’s Office Regional Administration and Local Government, and National Multi-sectoral Committee on Antibiotic Use and Resistance.

### Monitoring the implementation of actionable recommendations

A third workshop was convened in Dar es Salaam city to hear about the progress of implementation from the government and other implementing partners such as NGOs, professional associations and international organizations (e.g. UNICEF). In this workshop, stakeholders received updates from the government through the Committee of National Action Plan on Antimicrobial Resistance (NAPAR: 2017–22). During the third workshop, the government through NAPAR were already implementing one of the six recommendations of the ‘review of the 2017–22 NAPAR’ (the first recommendation). Additionally, the Government of Tanzania is formulating a policy on ‘health insurance for all’. Our study recommended that people should be sensitized to join health insurance schemes. It was realized that to take the other recommendations into action, more stakeholder engagement was needed. It was proposed that we should hold another stakeholders’ workshop, this time targeting donors.

## Lessons learnt from Tanzania

### Conflict of interest among stakeholders

During the workshop discussions, especially when the stakeholders were formulating actionable recommendations, there were always facilitator (in favour) and barrier (against) sides. However, conflict of interest among the stakeholders is something that needs to be clearly stated (noted) at the beginning of the IR process. For example, during the discussion on whether to include rapid diagnostic tests (RDTs) in the community pharmacies, there were conflicting interests between different professionals (pharmacy, medical and laboratory medicine). Some stakeholders thought that including RDTs in the community pharmacies was shifting the role of pharmacies from being dispensing outlets to health centres.

### Participation of stakeholders

Implementation of the PARAC project posed two challenges; when the same stakeholders were involved in the different workshops, the discussions became easier as the stakeholders had the knowledge on the discussed topic/subject. On the other hand, using the same stakeholders across different workshops ensured the bringing up of new and diverse ideas.

### Composition of the research team

The universities and research institutions may play the leading role, but the research team should be composed of a diverse number of stakeholders including those who are not representing academic and research institutions.^7^ For example, including policymakers and implementers at different levels of the health system as co-investigators during the implementation of the research^8^ will enhance the ownership of the research findings, which may ultimately facilitate the policy change and uptake.

### Funding of IR

With the current funding system, the maximum budget is planned before conducting research. However, IR is an iterative process that may need the revision of study protocol to accommodate the new things arising while conducting it or accommodate suggestions from the stakeholders. Securing an additional budget to implement a new proposed item may became challenging. The best approach for this is to include a reasonable percentage of contingency at the beginning of the process.

### Funding of an action plan

Traditionally, the funding of IR focuses on identification of relevant questions, subsequent development of an IR protocol and conducting the IR, analysis and write up of study results, and dissemination thereof. With strong and explicit support from Wellcome and UNICEF, timelines of the original study were extended, and the development of actionable recommendations and a prioritized action plan requested. This allowed greater uptake of the actionable recommendations into action plans and, in the future, their embedding into longer-term implementation plans. Another lesson learnt should be the ownership by the government mainly for the implementation of actionable recommendations.

## Conclusions

IR is a growing field when it comes to the Tanzanian context. Involvement of a range of stakeholders (government, academia, UN agencies, implementing partners) is crucial to develop IR capacity, and develop and execute a research agenda that is needs-based and responds to local implementation problems. IR funding, rather than stopping at dissemination of IR findings, needs to allow for the development of a subsequent action plan, which is based on prioritized actionable recommendations.

## Data Availability

Data used to draw this conclusion are available from the corresponding author under reasonable request.
